# A Predictive Model of Major Postoperative Respiratory Adverse Events in Pediatric Patients Undergoing Rigid Bronchoscopy for Exploration and Foreign Body Removal

**DOI:** 10.3390/jcm12175552

**Published:** 2023-08-25

**Authors:** Xiuwen Yi, Wenwen Ni, Yuan Han, Wenxian Li

**Affiliations:** Department of Anesthesiology, Eye & ENT Hospital of Fudan University, No. 83 Fenyang Road, Shanghai 200031, China; yixiuwen@eentanesthesia.com (X.Y.); niwenwen@eentanesthesia.com (W.N.)

**Keywords:** nomograms, LASSO regression, foreign body, respiratory tract diseases, anaesthesia, complications

## Abstract

**Background:** No nomogram has been established to predict the incidence of major postoperative respiratory adverse events (mPRAEs) in children undergoing rigid bronchoscopy for airway foreign bodies (AFB) removal and exploration of the airway, though some studies have confirmed the risk factors. **Methods:** 1214 pediatric patients (≤3 years old) undergoing rigid bronchoscopy for AFB from June 2014 to December 2020 were enrolled in this study. The primary outcome was the occurrence of mPRAEs, including laryngospasm and bronchospasm. Following that, a nomogram prediction model for the mPRAEs was developed. **Results:** The incidence of mPRAEs was 84 (6.9%) among 1214 subjects. American Society of Anesthesiologists physical status (ASA-PS), intraoperative desaturation (SpO_2_ < 90%), procedural duration and ventilatory approach were all independent risk factors of mPRAEs. The area under the receiver operating characteristic curve (AUC) value of the nomogram for predicting mPRAEs was 0.815 (95% CI: 0.770–0.861), and the average AUC for ten-fold cross-validation was 0.799. These nomograms were well calibrated by Hosmer-Lemshow (*p* = 0.607). Decision curve analysis showed that the nomogram prediction model is effective in clinical settings. **Conclusions:** Combining ASA-PS, intraoperative desaturation, procedural duration, and ventilatory approach, the nomogram model is adequate for predicting the risk of developing mPRAEs, followed by rigid bronchoscopy for AFB removal and exploration.

## 1. Introduction

Rigid bronchoscopy performed under general anesthesia is commonly performed for airway exploration and removal of the foreign body [1]. Anesthetic management could be challenging, especially in pediatric patients and toddlers aged 0–3 years, because of the shared airway. This can lead to a high occurrence of major postoperative respiratory adverse events (mPRAEs) like laryngospasm and bronchospasm, with severe rapid desaturation, hypoxic cardiac arrest or even death [2,3]. Therefore, there is a high need to identify the patients at an increased risk of developing these detrimental events to make the treatment plan more effective. Many studies focused on perioperative respiratory adverse events, but fewer studies have been done on postoperative respiratory adverse events [4,5,6]. Our study focuses more on adverse respiratory events, as postoperative laryngospasm or bronchospasm are more challenging to deal with and more likely to lead to severe consequences.

Several studies indicate that the risk factors regarding surgical factors, anesthetic induction and management, AFB type, intrinsic elements and the stability of pediatric patients all contribute to the mPRAEs. Anton-Pacheco JL et al. reported a longer procedural duration regarding the extraction of foreign bodies, or the patients of a relatively young age were at high risk of developing respiratory adverse events [7]. Meanwhile, the choice of anesthetic and ventilation mode may also be related to mPRAEs [8]. In this regard, a meta-analysis indicated that controlled ventilation reduces the occurrence of laryngospasm [9]. No nomogram has been established to predict the incidence of mPRAEs, although some studies have confirmed the risk factors of postoperative mPRAEs in children with AFB. However, it’s difficult to assess the occurrence of mPRAEs entirely by briefly relying on only a few risk factors, which may lead to misjudgment. Therefore, prediction models had a vital importance for accurately evaluating the mPRAEs.

Our study focuses more on preoperative and intraoperative factors of pediatric patients undergoing airway foreign body removal. We also proposed that the various factors may contribute to the occurrence of mPRAEs; following this, we developed a prediction model based on the LASSO machine learning method to provide a reference for anesthesiologists in predicting any risk factors which may lead to the occurrence of mPRAEs.

## 2. Methods

### 2.1. Study Design and Population

**Figure 1:**
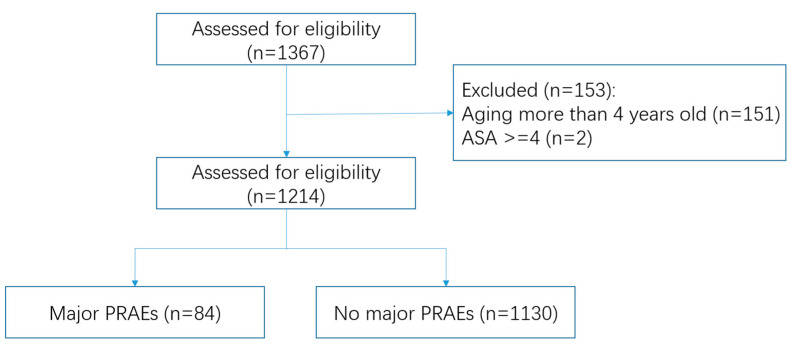
Numbers of participants enrolled and outcomes in the datasets.

This retrospective observational study was approved by the institutional research ethics committee of the Eye & ENT Hospital of Fudan University (Approval number: 2022109, Clinical Registration: ChiCTR2200064392), with informed consent to be obtained from all subjects involved in the study. The study adhered to the guidelines of Transparent Reporting of a multivariable prediction model for Individual Prognosis or Diagnosis (TRIPOD) [10]. To develop the prediction model, we analyze the data from all pediatric patients aged 0 to 3 years old who underwent AFB surgery under general anesthesia between June 2014 to December 2020 at the Eye & ENT Hospital of Fudan University. Exclusion criteria were ASA-PS of 4-6, missing data of mPRAEs (Figure 1). The pediatric patients with a high score of ASA-PS, with the occurrence of a vital emergency, differed from most children’s treatment plans in this study and were excluded.

Data were extracted every 24 h by Lex Clinical Data Application 3.2 (Hangzhou Lejiu Healthcare Technology Co., Ltd., Hangzhou, China.) from the EMR (electronic medical record) to a designated clinical data warehouse, including admission/transfer/discharge timings, laboratory orders/results, medication orders, administration events, flow sheet entries, the sequence of laboratory test and findings, performed procedures, medical reports, admission notes, treatment plans during the hospital stay, discharge sheet summary etc. All original data (i.e., pathology report, radiology report, progress notes, admit/discharge summary etc.) was sorted before the data analysis. Core elements of the data warehouse were de-identified entirely so that all queries and analytics could be carried out without exposing confidential health data and to maintain patient privacy.

### 2.2. Anesthetic Protocol

AFB removal operation with rigid bronchoscopy was performed under general anesthesia on all the patients with a routine anesthetic management protocol. The description of the protocol is defined as:

1. After entering the operating room, routine monitoring was performed for Electrocardiograph (ECG), blood pressure, and SpO_2_, followed by the introduction of inhaled anesthetic gas.
2. When the patient was slightly sedated and relaxed, peripheral venipuncture was performed, and the intravenous drug was injected to pursue anesthetic induction.
3. The ventilatory approach was based on the different conditions of children and the judgment of several senior anesthesiologists before surgery. If oxygenation were poor, the ventilatory approach would be changed accordingly.
  - The first was spontaneous respiration (Spont). Only sedatives and analgesics were used during the operation, not muscle relaxants. The anaesthesia was maintained by total intravenous anesthesia (TIVA) of propofol and remifentanil, depending on the individual’s respiratory rate.
  - The second was bronchoscopic lateral ventilation (BV), during which the sedative, analgesic and muscle relaxants were given intraoperatively. Oxygenation was maintained by intermittent manual ventilation through the lateral aperture by squeezing the reservoir bag. The anaesthesia was also maintained by total intravenous anesthesia (TIVA) of propofol and remifentanil, depending on the individual’s blood pressure and heart rate.
  - The third approach was manual jet ventilation (jet), in which sedatives, analgesics and muscle relaxants were also given intraoperatively, and children were given manual intermittent jet ventilation with the jet catheter tip placed 2 cm below the glottis. The anesthesia maintenance protocol was the same as above.
  - The fourth method was endotracheal tube ventilation (ETT), in which sedative, analgesic and muscle relaxant drugs were given intraoperatively, and children were ventilator-assisted through the endotracheal tube. The anesthesia maintenance protocol was the same as above.
4. After AFB was removed, the jet catheter would be replaced by the laryngeal mask airway immediately for recovery in the bronchoscopic lateral ventilation and manual jet ventilation groups, and the child would be placed in the lateral decubitus position simultaneously on the operating table. The child would be resuscitated in the operating room.
5. The children in spontaneous respiration and endotracheal tube ventilation groups would be resuscitated with mask ventilation and the original endotracheal tube without laryngeal mask airway replacement.
6. After removing the laryngeal mask or endotracheal tube, the children would be sent back to the ward when sufficient oxygenation could be maintained in the supine state with air inhalation.
7. Postoperative oxygen inhalation and nebulization therapy were given according to the patient’s condition.

### 2.3. Predictor Variables

Demographic, foreign body-related, symptom-related, imaging, surgical and anesthetic variables were recorded as candidate predictors. Demographic characteristics [gender, age, height, weight, body mass index (BMI)] were recorded using a standard protocol. Foreign body-related variables (AFB type, location of AFB, retention time of AFB) and symptom-related variables (cough, wheeze, asthma, stridor, dyspnea, cyanosis, fever) were derived from EMR. Imaging-related variables (Pneumonia, atelectasis, emphysema, pneumothorax) were identified through radiological examinations. Surgical-related variables (procedural duration) and anaesthetic-related variables (duration of anesthetic period, ASA-PS, ventilatory approach, change of ventilatory approach, intraoperative desaturation) were also recorded.

AFB type was defined as no AFB, organic and inorganic ones. The size of the foreign body was not considered because some foreign bodies may dissolve by the infiltration of secretions and be coughed out of the trachea through the coughing reflex. The location of AFB included the trachea, left bronchus, right bronchus, bilateral bronchus, and no foreign body. The retention time of AFB was divided into three layers, respectively ≤24 h, >24 h and ≤3 days, >3 days, just like the division method commonly used in previous studies [7]. The ventilatory approach was based on the anesthesiologist’s judgment. Change of ventilatory approach was defined as the patient needing to switch from one type of ventilation to another during the procedure. Intraoperative desaturation refers to SpO_2_ < 90% at any time during the procedure.

### 2.4. Primary Outcome

The outcome variable was defined as the occurrence of mPRAEs. Major PRAEs refer to laryngospasm and bronchospasm from the end of the procedure to back to the ward or Intensive Care Unit. Laryngospasm was defined as complete airway obstruction with associated rigidity of the abdominal and chest walls. Bronchospasm mean increased respiratory effort, particularly during expiration and wheezing on auscultation [3].

### 2.5. Statistical Analysis

The sample size for this prediction model was based on the theory of vanSmeden et al., which depended on the number of predictors, MAPE (mean absolute prediction error) and the proportion of the outcome event [11]. After preliminary data analysis, the incidence of the mPRAEs was 6.9% with four potential predictors, and the MAPE was set as 0.02, so the required sample size was 532. In our study, the number of enrolled patients was 1214, above the required sample size calculated.

Bivariate analysis was examined using the Mann-Whitney U test for continuous and Fisher’s exact chi-squared test for categorical variables. Samples with missing rates of less than 50% were imputed. Multiple imputations of missing data were processed with the chain equations [12]. All the collected data were used to develop the prediction model. LASSO logistic regression model was used to screen out the potential risk factors from the abovementioned variables. The LASSO can eliminate and reduce the impacts of comparatively irrelevant variables by constructing a penalty function with a hyperparameter λ to minimize their coefficients. Through 10-fold cross-validation, the λ that minimizes the mean squared error was chose, and the largest λ with a mean squared error within one standard deviation can also be chosen. For the final model, the variable corresponding to the optimal λ was selected. Then, the algorithm and corresponding intercept estimates were obtained from the maximum likelihood estimates according to the multiple logistic regression analysis, and the odds ratios and 95% confidence intervals (95% CIs) were calculated for these risk factors. A nomogram was constructed with the “rms” R package. The internal validation of the model was performed using 10-fold cross-validation without splitting the sample. Discrimination of the model was evaluated via the receiver operating characteristic (ROC) curve and the AUC. The model’s calibration was assessed by the Hosmer-Lemeshow test and was visualized through the calibration curve. Decision curve analysis was conducted to determine the clinical usefulness of the nomogram by quantifying the net benefits at different threshold probabilities in the dataset. All statistical tests were two-sided, and *p* < 0.05 was considered significant. LASSO feature selection was applied with the “glmnet” R package. Statistical analysis was performed in R version 3.6.1 (R Foundation for Statistical Computing) and STATA software version 15.0 for Windows (StataCorp, College Station, TX, USA).

## 3. Results

### 3.1. Baseline Characteristics

The flow chart of the study design is shown in Figure 1. A total of 1367 children were enrolled in the study. Among those, 1214 pediatric patients fulfilled the inclusion criteria. In the cohort, 6.9% (*n* = 84) of patients presented with mPRAEs, including 28 incidents of bronchospasm and 56 laryngospasms. Patient characteristics in the dataset are given in (Table 1).

### 3.2. Development of the Model

**Figure 2:**
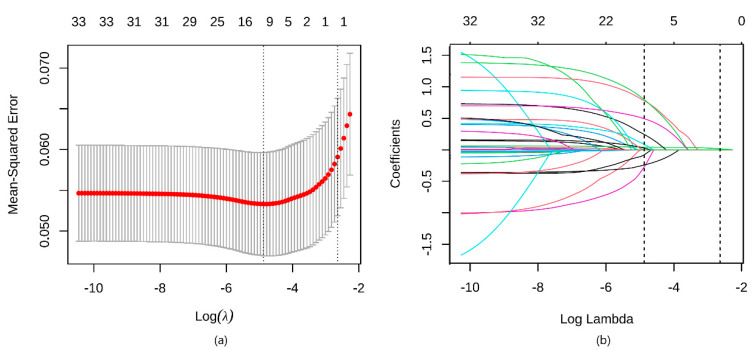
Predictor selection using LASSO binary logistic regression model. (**a**) Identification of the optimal penalization coefficient lambda (λ) in the LASSO model used ten−fold cross−validation and the minimum criterion. A vertical dotted line was drawn at the value selected using ten−fold cross−validation, where optimal values were by using the minimum criteria and the 1 standard error of the minimum criteria (the 1−se criteria). The meaning was the same as the dotted line in (**b**). (**b**) LASSO coefficient profiles of the features. Each color line represented the value taken by different coefficients in the model. Lambda (λ) was the weight given to the regularization term (the L1 norm). We would keep the variable whose coefficient was not 0 for the target λ value.

**Figure 3:**
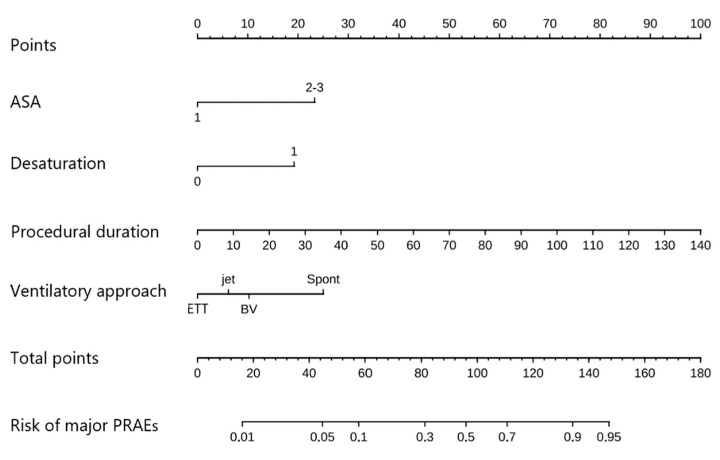
Nomogram to estimate the probability of mPRAEs after rigid bronchoscopy for AFB diagnosis and removal. A nomogram for mPRAEs was developed and integrated with the predictors. Find the predictor points on the uppermost point scale that correspond to each patient variable and add them up. The total points projected to the bottom scale indicate the probability of mPRAEs. ETT, endotracheal tube; jet, manual jet ventilation; BV, bronchoscopy ventilation; Spont, spontaneous respiration.

LASSO regression analysis proposed that procedural duration, ASA-PS, ventilatory approach, and intraoperative desaturation could be identified as predictors (Table 2). The process of variable selection by LASSO regression is shown in (Figure 2). The model incorporating the above independent predictors is developed and presented as the nomogram (Figure 3).

### 3.3. Validation of the Model

#### 3.3.1. Discrimination

**Figure 4:**
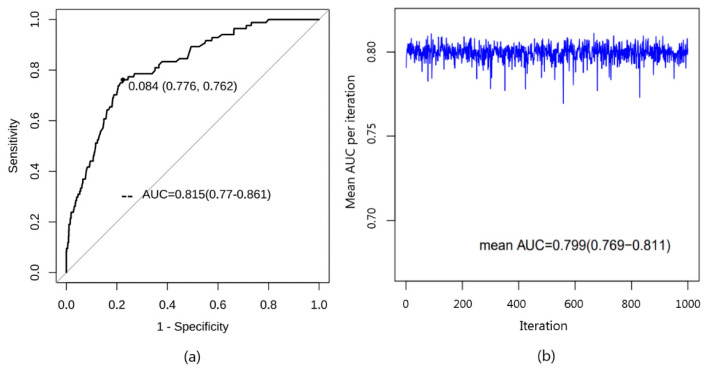
(**a**) Receiver operating characteristic (ROC) curve of the nomogram. The nomogram had good discriminative power with an AUC (95% confidence interval) of 0.815 (95% CI: 0.770~0.861) in the dataset. The optimal inflection point was 0.084, and the corresponding 1-specificity and sensitivity were 0.776 and 0.762. (**b**) The mean AUC obtained by ten-fold cross-validation was 0.799 (95% CI: 0.769~0.811). The *x*-axis represented ten-fold cross-validation was performed 1000 times. The *y*-axis represented the average AUC for each ten-fold cross-validation.

The AUC is plotted in (Figure 4). This model has excellent discriminative power with an AUC of 0.815 (95% CI: 0.770–0.861) in the dataset (Figure 4a). The model’s ROC curve analysis shows that the predicted value’s best cut-off point is 0.084, according to the Youden index (Figure 4a). As internal validation, ten-fold cross-validation shows that the average AUC of the model is high (mean AUC = 0.799 (95% CI: 0.769–0.811)), indicating that it has good discrimination (Figure 4b).

#### 3.3.2. Calibration

**Figure 5:**
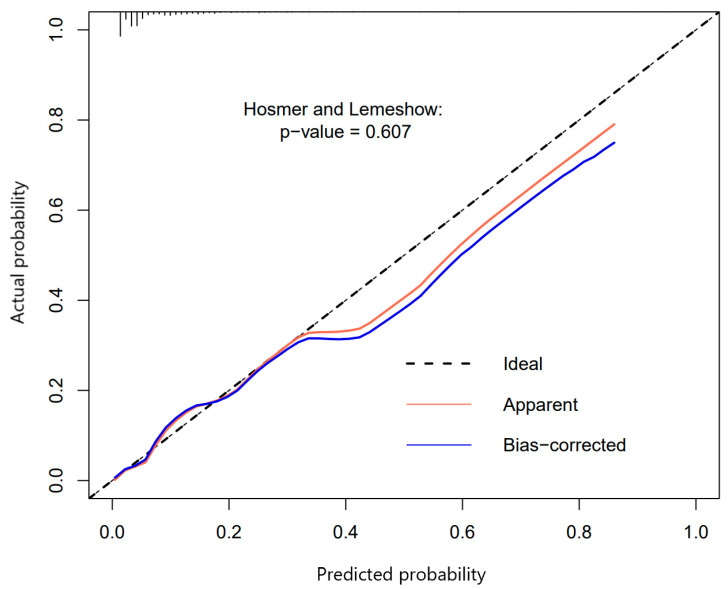
The calibration curve of the nomogram for predicting mPRAEs in the dataset (*p* = 0.607). The *y*-axis represented the actual rate of mPRAEs. The *x*-axis represented the predicted probability of mPRAEs. For a nomogram with well-calibrated, the scatter points should be arranged along a 45-degree diagonal line. The data was concentrated in the low-probability region, and the model was consistent when the probability was low.

The calibration curve was plotted in (Figure 5), which was evaluated with the Hosmer-Lemeshow test in the dataset (*p* = 0.607). When the probability of the prediction model is low, it shows a high consistency, which is consistent with our data. This model was well calibrated, with no significant difference between the predicted and the actual probability. The sensitivity and specificity of the prediction model for mPRAEs were respectively 0.762 (0.671–0.853) and 0.776 (0.752–0.800). And the positive predictive value and negative predictive value were respectively 0.202 (0.158–0.246) and 0.978 (0.968–0.987). The positive and negative likelihood ratios were respectively 3.403 (2.896–3.999) and 0.307 (0.209–0.450).

#### 3.3.3. Clinical Use

**Figure 6:**
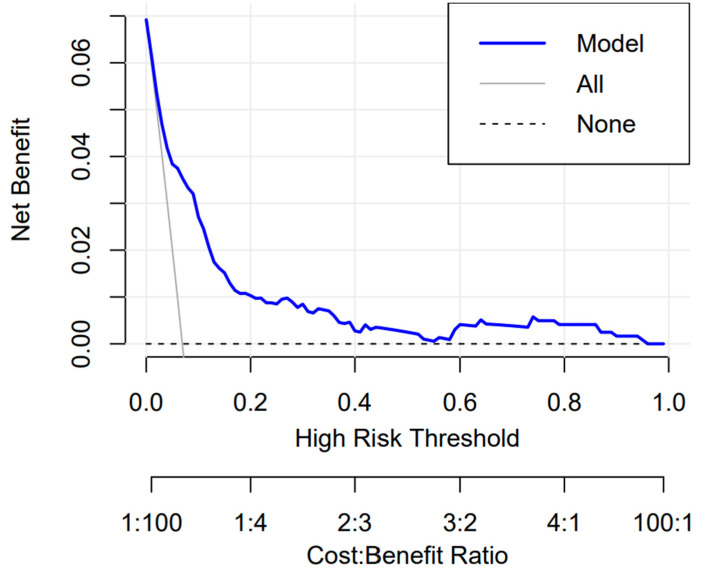
Decision curve analysis for the nomogram. The *y*-axis measured the net benefit. The blue line represents the nomogram. The grey line represented the assumption that all patients have suffered mPRAEs, with all receiving the interventions, and the net benefit was a backslash with a negative slope. The dotted line represented the assumption that no patients had suffered mPRAEs, had no one interventions, and the net benefit was 0. The dataset’s curve was above the extreme curves within a large threshold range (10–70%), indicating that the model had a high benefit rate.

The model’s decision curve analysis (DCA) is presented in (Figure 6). We did DCA on our prediction model to assess the net benefit that patients could receive. As the decision curve indicates, the model has an apparent net benefit for almost all threshold probabilities, especially in 10–70% thresholds (Figure 6). However, if the threshold probability were less than 10%, the net benefit of the nomogram was equivalent to predicting positive results for all patients.

## 4. Discussion

In the present study, the incidence of mPRAEs is 6.9%, lower than the 17.6% and 17.4% rates reported in the other two retrospective studies of airway foreign bodies in China [2,13]. This may be related to the fact that the medical centre of this study is a specialized hospital for Eye and ENT cases and has more operations of ARB removal and experience in anesthesia management. The procedural duration, ASA-PS, ventilatory approach, and intraoperative desaturation were identified as the risk factors for predicting mPRAEs in AFB removal in children. Pediatric patients with longer procedural duration, higher ASA-PS, preserved spontaneous respiration, and intraoperative desaturation is more likely to induce post-operation laryngospasm and bronchospasm. Furthermore, the prediction model of the nomogram is established. It is well-calibrated and discriminated for individualized prediction and facilitates individual treatment. Our model will benefit the clinician in making a more suitable surgical and anesthetic plan by improving the preoperative pulmonary condition of the children, using controlled ventilation, and reducing the procedural duration. For example, if the child’s ASA-PS is 2 or 3, and the procedural duration is expected to be long, desaturation could occur during the operation, and the anaesthetist may have proceeded with a pre-surgery plan for the ventilation approach. As the occurrence probability of the mPRAEs can be found in the nomogram of this study, the corresponding predictive factors would help in designing a more accurate approach pre-surgery, such as shortening the procedural duration as much as possible, using bronchoscopy ventilation, manual jet ventilation, and ETT to maintain intraoperative oxygenation and prevent intraoperative desaturation, thereby reducing the probability of mPRAEs in children. If none of the above intraoperative predictors can be changed, it can also guide us to make adequate preparations during recovery to prevent mPRAEs.

AFB removal in infants has a high incidence of RAEs and often leads to severe consequences, such as suffocation, cardiac arrest, and rare cases of mortality [14,15,16]. However, developing an appropriate and optimal anesthetic plan for these children with complex respiratory conditions and severe pulmonary complications is hard. Moreover, the prognosis of children may be affected by many risk factors. Therefore, this study’s prediction model helps solve these problems.

Previous reports have elucidated the risk factors for the prognosis of children with AFB, including retrospective studies, meta-analyses, and reviews. Some studies have found that the retention time of AFB with prolonged procedural duration could cause perioperative complications in children [13,17]. Various meta-analyses and reviews reported laryngospasm and bronchospasm had a lower incidence when controlled ventilation was performed [9,18,19]. Additional clinical studies were required to substantiate this issue. However, the present study found that procedural duration, ASA-PS, ventilatory approach, and intraoperative desaturation were independent risk factors for predicting laryngospasm and bronchospasm in pediatric AFB removal, which was constant with the findings of studies being done previously.

The results show that intraoperative desaturation is a reliable predictor for mPRAEs. As we all know, desaturation often occurs along with laryngospasm and bronchospasm or attributes to them [20,21]. We also found that airway obstruction and respiratory infection may cause desaturation. Airway obstruction is a common risk factor for laryngospasm and bronchospasm, consistent with previous studies [22,23]. Foreign bodies stranded in the bronchus for a long time often cause respiratory tract infection, which is one of the risk factors for laryngospasm and bronchospasm [21,24]. Therefore, desaturation resulting from airway obstruction and respiratory disease is also associated with mPRAEs.

This model also indicates that ASA-PS 2 and 3 may be risk factors for laryngospasm and bronchospasm, confirmed by previous studies [4,25]. They also show that ASA-PS is related to high incidences of laryngospasm and bronchospasm and is a variable contributing to the condition [21]. Our validated nomogram indicates that the children’s severe condition may lead to laryngospasm and bronchospasm. For these children, effective preventive measures should be taken to reduce the occurrence of laryngospasm and bronchospasm.

In addition, variables related to procedural duration and ventilatory approach seem to have a connection with a high incidence of laryngospasm and bronchospasm. The longer the surgical procedure duration, the higher the incidence of laryngospasm and bronchospasm, similar to the conclusion of Yu Cui et al. and Maddali et al. [13,26]. Compared to spontaneous breathing, bronchoscopy ventilation, manual jet ventilation, and controlled ventilation with endotracheal tube predict lower incidences of laryngospasm and bronchospasm. This is somewhat similar to the theory of Yuqi Liu et al., which showed a lower incidence of laryngospasm and bronchospasm with controlled ventilation [9]. It might be challenging to reach a suitable sedation level to prevent harmful reflexes, such as laryngospasm, bronchospasm, body movement or bucking, and simultaneously maintain spontaneous respiration [27]. Maintaining a deeper depth of anesthesia in a controlled ventilation group is often necessary; this precaution may lead to fewer mPRAEs.

In recent years, other risk factors or predictors of laryngospasm and bronchospasm have been identified, such as age, retention time of AFB, AFB type, and preoperative pneumonia [7,13,21]. These factors are also included in our study. Still, they are not turned out to be the independent risk factors due to the different definitions of the primary outcome, the different age ranges of the enrolled children, and the difference in science popularization for foreign body aspiration in the respective study location.

This study is one of the first to establish a predictive model for postoperative complications of AFB surgery according to preoperative and intraoperative factors, which allows anesthesiologists to evaluate the risks of complications and actively take preventive measures to avoid adverse events in children. The AUC of the model (0.815) and the *p* value of the Hosmer Lemeshow test (0.607) show that the model has excellent discrimination and calibration. The prediction model’s positive likelihood ratio (3.403) and negative likelihood ratio (0.307) show good accuracy.

DCA is an effective method to evaluate the clinical benefit of the model [28,29]. The horizontal line in the graph shows that if all the samples are negative with no mPRAEs, the net benefit is 0. The diagonal line indicates that all samples are positive, all occurred mPRAEs, and the net benefit is a backslash with a negative slope. The curve from the model is compared with these two lines. The curve of the training set is higher than the extreme curves within a large threshold range, especially in threshold probabilities of 10–70%, indicating that the model has an obvious net benefit rate and good fitting. The decision curve showed that if the threshold probability of a patient or doctor is >10%, using the nomogram in the current study to predict the rate of mPRAEs adds more benefit than the all-occurred-mPRAEs scheme or the none-occurred-mPRAEs scheme.

This study has several limitations. First, this study is a retrospective study based on the extraction of database information, and there could be a presence of recorded bias. Then, no external validation was performed, which could be supplemented in our future studies. However, the sample size of our research is significant, and ten-fold cross-validation indicated it might provide a good validation effect. Anesthesiologists were not included in the model as a risk factor, which is one of the limitations of this study. Several anesthesiologists decided on each child’s ventilation plan before surgery, which may have little impact on mPRAEs.

## 5. Conclusions

In summary, we found that procedural duration, ASA-PS, ventilatory approach and intraoperative desaturation were identified as the risk factors of mPRAEs during AFB removal. Further, we developed a predictive model and nomogram, verified in predicting mPRAEs. It could facilitate the individualized prediction of mPRAEs of children undergoing rigid bronchoscopy for AFB diagnosis and removal.

## Figures and Tables

**Table 1:** Bivariate analyses of study variables versus mPRAEs for training dataset.

Variables	Non-Major PRAEs Group (*n* = 1130)	Major PRAEs Group (*n* = 84)	Z Value	*p* Value
Gender, *n* (%)			0.161	0.688
Male	771 (68.2)	55 (65.5)		
Female	359 (31.8)	29 (34.5)		
Age, Median (IQR) (months)	18 (14, 23)	17.5 (14, 21)	0.929	0.335
Duration of anesthesia, Median (IQR) (min)	24 (18, 33)	46 (32.5, 68)	105.28	<0.001
AFB type, *n* (%)			1.557	0.459
No AFB	77 (6.8)	3 (3.6)		
Inorganic	8 (0.7)	1 (1.2)		
Organic	1045 (92.5)	80 (95.2)		
Cough, *n* (%)			0.546	0.289
No	30 (2.7)	4 (4.8)		
Yes	1100 (97.3)	80 (95.2)		
Asthma, *n* (%)			5.722	0.017
No	720 (63.7)	42 (50)		
Yes	410 (36.3)	42 (50)		
Dyspnea, *n* (%)			0.239	0.625
No	995 (88.1)	76 (90.5)		
Yes	135 (11.9)	8 (9.5)		
Wheeze, *n* (%)			1.812	0.331
No	1115 (98.7)	82 (97.6)		
Yes	15 (1.3)	2 (2.4)		
Cyanosis, *n* (%)			0.235	0.628
No	1046 (92.6)	76 (90.5)		
Yes	84 (7.4)	8 (9.5)		
Stridor, *n* (%)			1.124	0.7
No	1106 (97.9)	82 (97.6)		
Yes	24 (2.1)	2 (2.4)		
Fever, *n* (%)			0.041	0.84
No	945 (83.6)	69 (82.1)		
Yes	185 (16.4)	15 (17.9)		
Location of AFB, *n* (%)			8.609	0.072
No AFB	137 (12.1)	5 (6)		
Bilateral bronchus	10 (0.9)	2 (2.4)		
Right bronchus	419 (37.1)	31 (36.9)		
Left bronchus	466 (41.2)	43 (51.2)		
Main trachea	98 (8.7)	3 (3.6)		
Emphysema, *n* (%)			0.155	0.694
No	408 (36.1)	28 (33.3)		
Yes	722 (63.9)	56 (66.7)		
Atelectasis, *n* (%)			0.024	0.877
No	1022 (90.4)	75 (89.3)		
Yes	108 (9.6)	9 (10.7)		
Pneumonia, *n* (%)			5.802	0.016
No	854 (75.6)	53 (63.1)		
Yes	276 (24.4)	31 (36.9)		
Pneumothorax, *n* (%)			3.386	0.302
No	1126 (99.6)	83 (98.8)		
Yes	4 (0.4)	1 (1.2)		
Weight, Median (IQR) (kg)	12 (10, 13)	11.3 (10, 12.6)	1.94	0.164
Height, Median (IQR) (cm)	83 (80, 90)	80 (77.5, 86)	5.425	0.02
BMI, Median (IQR) (kg/m^2^)	16.9 (15.4, 18.8)	17.4 (16.0, 18.8)	2.913	0.088
Ventilatory approach, *n* (%)			27.217	<0.001
Spontaneous respiration	175 (15.5)	30 (35.7)		
Bronchoscopy ventilation	125 (11.1)	4 (4.8)		
Manual jet ventilation	752 (66.5)	41 (48.8)		
Controlled ventilation by ETT	78 (6.9)	9 (10.7)		
Change of ventilatory approach, *n* (%)			87.231	<0.001
No	1111 (98.3)	67 (79.8)		
Yes	19 (1.7)	17 (20.2)		
ASA-PS, *n* (%)			27.66	<0.001
1	484 (42.8)	12 (14.3)		
2	580 (51.3)	62 (73.8)		
3	66 (5.8)	10 (11.9)		
Procedural duration, Median (IQR) (min)	14 (9, 21)	20 (14, 35.5)	40.428	<0.001
Retention time of AFB, *n* (%)			0.875	0.646
≤24 h	307 (27.2)	22 (26.2)		
24 h~3 days	381 (33.7)	25 (29.8)		
>3 days	442 (39.1)	37 (44)		
Intraoperative desaturation, *n* (%)			42.083	<0.001
No	876 (77.5)	38 (45.2)		
Yes	254 (22.5)	46 (54.8)		

AFB, Airway foreign body; ASA, American Society of Anesthesiologists; ETT, endotracheal tube; BMI, body mass index; IQR, interquartile range.

**Table 2:** Parameters of multivariate logistic regression model.

Predictors	Estimate	*p* Value	OR (95% CI)	Wald
(Intercept)	−4.079	<0.001	0.017 (0.007–0.036)	0
Procedural duration	0.041	<0.001	1.042 (1.028–1.057)	1.086
Bronchoscopy ventilation	−1.439	0.021	0.237 (0.059–0.717)	0.056
Manual jet ventilation	−1.086	<0.001	0.338 (0.199–0.578)	0.114
Controlled ventilation by ETT	−0.85	0.106	0.427 (0.141–1.133)	0.183
ASA-PS 2-3	1.34	<0.001	3.821 (2.011–7.909)	14.598
Intraoperative desaturation	1.105	<0.001	3.019 (1.829–4.979)	9.114

ASA, American Society of Anesthesiologists; ETT, endotracheal tube.

## Data Availability

The data that support the findings of this study are available from the corresponding author [W.L.], upon reasonable request.
